# The Mediation Role of Perceived Benefits and Barriers in the Relationship Between Support Provided by Significant Others and Physical Activity of Adolescents

**DOI:** 10.1177/00315125231151780

**Published:** 2023-01-09

**Authors:** Filipe Rodrigues, Diogo Monteiro, Vítor P. Lopes

**Affiliations:** 1 ESECS, Polytechnic of Leiria, 70867 , Leiria, Portugal; 2 Life Quality Research Center, Rio Maior, Portugal; 3 70863 Instituto Politécnico de Bragança, Campus de Santa Apolónia, Bragança, Portugal; 4 Research Center in Sports Sciences, Health Sciences and Human Development (CIDESD), Vila Real, Portugal

**Keywords:** best friend, parents, barriers, benefits, physical activity, sex difference

## Abstract

We investigated whether the relationship between significant others’ social support and adolescents’ physical activity (PA) is mediated by perceived barriers and benefits of PA. In this cross-sectional study, we analyzed data from 497 adolescents (girls = 272, boys = 225) aged between 12-18 years (*M* = 15.87, *SD* = 1.43) from six different middle and secondary schools. We collected data regarding social cognitive variables and PA with self-report measures and calculated the metabolic equivalent of total amount PA. We performed structural equation modeling and mediation analyses and found our proposed models fit the data. In girls, perceived PA benefits mediated the association between support provided by friends (β = .13; IC 95% = .02 .29), a best friend (β = .14; IC 95% = .03, .33), and parents (β = .07; IC 95% = .01, .18), and PA. Similarly in boys, perceived PA benefits partially mediated the association between support provided by parents (β = .09; IC 95% = .04, .37), friends (β = .11; IC 95% = .05, .40), and a best friend (β = .10; IC 95% = .05, .40) and PA. Perceived barriers to PA did not display any significant mediation role for either sex. Interventions to foster others’ support for PA, especially from a best friend, are important for promoting PA among adolescents.

## Introduction

While the physiological and psychological benefits of physical activity (PA) are well documented (Janssen & LeBlanc, 2010; Reiner et al., 2013; WHO, 2014), most adolescents do not engage in sufficient PA to obtain its associated benefits (Directorate-General for Education Youth Sport and Culture, 2018; van Sluijs et al., 2021). Monteiro et al. (2021) showed that social support provided by significant others influenced PA engagement, but it is still not clear which relationships with significant others may be most important for promoting more PA, particularly since adolescents experience shifts in their social interactions as they mature (Lopes et al., 2013).

### Social Support From Significant Others and Perceived PA Barriers and Benefits

Social cognitive theory (Bandura, 1997; Glanz et al., 2015) has defined social support as a factor related to the approach-avoidance action towards a given behavior. By acting as a cognitive factor for positive health behaviors, social support can produce a health benefit effect (Uchino et al., 1996) and can be carried out in several ways, such as emotionally, instrumentally, and informationally (Birch, 1998). In adolescence, girls and boys that are supported by significant others to be physically active have tended to display adaptive outcomes, such as greater self-esteem and a greater amount of PA (Lopes et al., 2015; Monteiro et al., 2021).

Social cognitive theory also accounts for crucial concepts of motivation such as perceived benefits and barriers to a given behavior (Bandura, 1997, 2004; Glanz et al., 2015). Self-efficacy operates together with goals, outcome expectations, and perceived environmental barriers and facilitates the regulation of human motivation, behavior, and well-being (Bandura, 2004). According to Williams et al., (2005), perceived benefits and barriers of PA are related to its outcome expectancy; this is a central construct in the social cognitive model of health behavior that should be interpreted as a factor in positive and negative outcome expectations. The perception of benefits of PA, such as improving mental and physical health, weight management, reduction of cardiometabilc disease, building bones and muscles strength, and improving the ability to do everyday activities can lead adolescents to be more active and to engage in PA, exercise, or sports (Lopes et al., 2015). On the other hand, perceived barriers of PA such as lack of time, lack of motivation, fear of injury, a lack of self-efficacy, and a lack of support and resources (e.g., exercise equipment) can lead to decreased PA (Cheng et al., 2003; van Sluijs et al., 2021).

There is some evidence (Ginis et al., 2011; Li et al., 2012) that social support predicts PA, and that outcome expectations (i.e., barriers and benefits) may mediate between social support and PA. However, prior studies have mainly tested this mediation hypothesis in the adult population, which is significantly different from adolescents who are still amidst self-discovery and personal growth (Ginis et al., 2011; Li et al., 2012). In addition, other studies have only examined the associations between support provided by physical education teachers and intentions to be physically active, without measuring the behavior itself (Cid et al., 2019; Rodrigues et al., 2020). Support from friends (Mendonca et al., 2014) and a best friend (Monteiro et al., 2021) has been significantly associated with PA. Hsu et al. (2011) examined associations between support provided by friends and family for engaging in PA and negative meanings of PA. While these results showed significant associations between the support provided by family members and perceptions of the importance of vigorous-intensity PA, the possible influence of perceived benefits and barriers in this association was not considered.

A recent systematic review of PA support literature demonstrated that the relative importance of different sources and types of social support for PA vary with the characteristics of the particular support person(s) (King et al., 2008). For example, social support provided by parents is usually in the form of transportation to sporting venues, sports equipment, and reinforcement and encouragement to engage in leisure-time PA; whereas friends generally provide support by joining in more vigorous PA and competitive sports (Lopes et al., 2013). There is also evidence that certain types of social support, such as engaging in activities with the best friend are more strongly associated with leisure-time PA (Monteiro et al., 2021), whereas other types of support, such as encouragement, have been important for active commuting (King et al., 2008). Hence, it seems possible that the social support provided by significant others tends to demonstrate and ackwnoledge the benefits of PA and encourage an adolescent to engage in this behavior. As a consequence, greater perceived benefits of PA should be associated with greater PA engagement; and, on the other hand, more perceived barriers (e.g., the lack of encouragement towards exercise or sports) based on the support provided by significant others should be associated with lower PA engagement.

### Current Research

In this study, we acknowledged the specific types of social support provided by certain significant others for adolescents. If boys and girls are generally assumed to engage in regular PA for mostly self-determined reasons (Rodrigues et al., 2020) or to obtain higher grades in physical education (PE) (Cid et al., 2019), we might assume that being physically active or not is independent of the support provided by significant others. However, in the present study, we expanded this area of research with social cognitive models in complement with some previous literature (Lopes et al., 2015; Monteiro et al., 2021) by investigating how the support provided by parents, friends, and a best friend might be differentially associated with perceived benefits and barriers of PA, and, consequently, with the total amount of PA.

Significant others, including friends, best-friends, and parents are all factors that could contribute to a pleasant PA experience. We differentiated general friends from a person’s best friend; we defined friends as a more general representation of relationships and defined the best friend as a person an individual values above other friends (Lopes et al., 2013). Furthermore, a best friend represents a higher level of relationship (e.g., social intimacy, deeper connection, highly valued) among a general set of friends or even peers. While adolescents could have several best friends, for the purposes of this study, we only considered the adolescent’s closest friend – the one with the highest degree of relationship. Friends are typically similar on a wide range of characteristics, such as gender, age, socio-economic background, attitudes and interests (Bot et al., 2005; Lopes et al., 2015). Although social support from friends and the best friend has been generally identified as a positive PA correlate (Sawka et al., 2013), research about the role of friendships in PA engagement has been limited.

Understanding friend relationships more comprehensively could better inform caring adults as to how to best design efficient interventions for promoting PA among boy and girl adolescents. We expected this study to provide significant information on how to target PA interventions for this population, since inactive girl and boy adolescents are at risk of being physically inactive in adulthood (Lopes et al., 2013). Knowledge gained from this research might guide PE teachers in their choices of intervention during class sessions and might make them aware of the importance of significant others for promoting supportive communications within the family that provide an essential factor to the adolescent’s well-being, with the ultimate goal of encouraging adolecents to overcome barriers and beter focus on benefits to engaging in PA (Monteiro et al., 2021).

In the present study we aimed to examine the association between support provided by the best friends, friends, and parents, barriers and benefits of PA, and PA in a sample of girl and boy adolescents. Considering previously cited literature, we hypothesized that support from parents, friends, and the best friend are positively related to benefits, and negatively associated with barriers. Specifically, we speculated that the support provided by the best friend would be the most significant determinant of perceived benefits and the amount of PA, as support from a best friend is considered a fundamental motivator toward well-being in adolescence (Monteiro et al., 2021). We also hypothesized that benefits of PA would be positively associated with PA, whereas barriers would be negatively associated with PA (Cheng et al., 2003). Finally, based on past research showing how perceptions of social support may influence PA engagement (Lopes et al., 2013), we expected that the indirect associations between social support provided by significant others and PA levels via benefits of PA would be significant, but the perceptions of barriers of PA would be less important. The novelty of this research is twofold. First, we examined support provided by three different significant groups of persons that can influence adolescent behavior. Second, our analysis of any mediating role of perceived benefits and barriers of PA is new. While we expected that the social support could directly influence PA, the perception of benefits and barriers could mediate this analysis by increasing the power of specific social support in explaining PA.

## Method

### Participants and Procedures

We used an a priori sample size calculator for structural equation model analysis (Soper, 2022) to calculate the minimum required sample size for this study to be valid and reliable. The following inputs were used: anticipated effect size = .03 (medium effect); desired statistical power = .95; number of latent variables = 5; number of observed variables = 1; probability level = .05. The results suggested a minimum of approximately 223 participants, suggesting that our sample size was sufficiently large.

Adolescents (*n* = 497; girls = 272, boys = 225) aged between 12-18 years (*M* = 15.87; *SD* = 1.43) from six middle and secondary schools completed a self-report questionnaire to generate data. To be eligible for this study, potential participants needed to be aged between 12-18 years, corresponding to the adolescence age period (Sacks, 2003).

Parents or tutors had to sign informed consent before their child could participate in this study. Adolescents also gave their written assent before filling out the questionnaires. Following ethical institutional approval for this study by the Research Center in Sports Sciences, Health Sciences and Human Development, we contacted several school principals and directors. After board approval and before class, we gave a brief explanation of the study purpose, and we provided comfortable conditions to participants for the completion of the questionnaires. Questionnaires were completed using the paper-and-pencil method. Mean time taken to complete the survey was approximately 12 minutes.

### Instruments

We measured adolescent perceptions of social support provided by the best friend, friends, and parents with the Friend Support Scale (Jago et al., 2012). Three stems were created according to the support group, as followed: “*how often do your best friend?*”; “*how often do your friends?* and “*how often do your parents?*” To differentiate the best friend from friends in general, all participants were asked to identify their best friend within the school. Regarding the perception of social support provided by friends in general, participants were asked to report their overall perception of their friends. Participants provided responses to these four statements: (a) encourage you to exercise or play sports; (b) exercise or play sports with you; (c) tell you that you are doing well in exercise or sports and (d) watch you take part in exercise or sports. All items were answered on a four-point scale ranging between 1 (*strongly disagree*) and 4 (*strongly agree*). The friend support scale has been used previously in other studies where it has shown acceptable scores of validity and reliability for adolescents of the same age and language group (Lopes et al., 2015; Monteiro et al., 2021).

We measured the participants’ perceived benefits and barriers of PA with the Exercise Benefits-Barriers Scale (Sechrist et al., 1987). This version contains 43-items grouped into two factors, namely barriers (14 items: item example “*Exercising takes too much of my time*”) and benefits (29 items: item example “*I enjoy exercise*”) in which participants responded to each item using a four-point scale ranging from 1 (*strongly disagree*) to 4 (*strongly agree*).

We used the International Physical Activity Questionnaire short version for adolescents (Hagströmer et al., 2008), a self-report measure, to assess PA, considering leisure time PA, household activities, school-related PA, and commuting. PA frequency and duration were measured in days per week and time per day, respectively. Last, we calculated the metabolic equivalent (MET) of the task by considering the following equation: Walking: MET-min · week−1 = 3.3 × walking minutes × walking days; Moderate: MET-min week−1 = 4.0 × moderate-intensity activity minutes × moderate days; Vigorous: MET-min · week−1 = 8.0 × vigorous-intensity activity minutes × vigorous-intensity days.

### Statistical Analysis

#### Preliminary Analyses

Missing data were handled by the multiple imputation procedure. Descriptive statistics such as range, means and standard-deviations, skewness, and kurtosis were calculated. To determine the statistical significance of any deviation from normal distribution, the skewness and kurtosis estimates were divided by their corresponding standard error to get the z score. Z-score that fell below |1.96| suggested a normal distribution. Alpha coefficients for internal consistency were calculated considering as acceptable coefficients ≥0.70. We also evaluated bivariate and partial correlations of the variables under analysis. Partial correlations were used to control for age. We set statistical significance at *p* < 0.05. We analyzed data using the Statistical Package for the Social Sciences (SPSS, Version 25.0., IBM Corp., Armonk, NY, USA).

#### Structural Equation Modelling Analysis

We performed structural equation modelling to test proposed associations, considering the maximum likelihood robust estimator, since it is robust against non-normal data, using Mplus 7.3 (Muthen & Muthen, 2010). We verified our analysis of model fit through the traditional goodness-of-fit indexes, namely: Comparative Fit Index (CFI); Tucker-Lewis Index (TLI); Standardized Root Mean Square Residual and Root Mean Square Error of Approximation (RMSEA) and its respective Confidence Interval (CI) at 90%. The following cut-off values were considered (Hair et al., 2019), namely: CFI and TLI ≥ 0.90, and SRMR and RMSEA ≤ 0.08. Direct and indirect between variables were assessed considering standardized beta coefficients. Significance was set at CI 95% (Williams & MacKinnon, 2008).

#### Mediation Analyses

Mediation analysis using SPSS PROCESS (Version 3.3, IBM Corp., Armonk, NY, USA) was performed to estimate the effects within the hypothesized mediation effects of PA benefits and barriers. Predictor variables, mediators, and outcome variables were standardized before testing serial mediation analysis as proposed by several authors (Hayes, 2018; Preacher & Hayes, 2008). Support provided by significant others, barriers and benefits were employed independently in the model as a manifest variable, computed by calculating the mean of the four scale items. Based on mediation analysis assumptions (Hayes, 2018), a sequential mediation model was tested (model 4; one serial mediation) in which one mediators was defined, in order to examine the associations among variables of interest. Specifically, predictor variable (e.g., support provided by the best friend), outcome variable (i.e., total amount of PA), and one serial mediators (e.g., benefits) were imputed in the mediation model for analysis. Bootstrap with 5000 samples was employed, and the confidence interval at 95% was considered for significance (Williams & MacKinnon, 2008). Bootstrapping procedures allowed for re-sampling as recommended for mediation analysis, particularly when based in ordinary least squares (OLS) calculations (Hayes, 2018).

## Results

### Preliminary Analyses

Descriptive statistics and internal consistency coefficients are shown in Table 1. Mean scores for support provided by the best friend were greater compared to other support sources for girls. For boys, support provided by friends also displayed the greatest mean scores compared to other forms of social support. PA scores were a non-normal distribution and were thus handled using the Kruskall-Wallis test. Boys showed a statistically (*p* < .05) greater amount of total PA compared to girls. Internal consistency coefficients were all above cutoff, displaying latent factor reliability.

**Table 1.** Descriptive Statistics of Participants and Internal Consistency Coefficients.

	Girls sample						Boys sample					
	Range	*M*	*SD*	S	K	IC	Range	*M*	*SD*	S	K	IC
Support best friend	1–4	3.07	.75	−.82	.40	.87	1–4	3.11	.77	−.90	.40	.88
Support friends	1–4	3.06	.61	−.53	.68	.81	1–4	3.24	.66	−1.06	1.48	.84
Support parents	1–4	2.68	.74	−.10	−.62	.81	1–4	2.78	.75	−.18	−.59	.77
Benefits	1–4	3.19	.44	−.22	.40	.92	1–4	3.37	.49	−.67	1.29	.93
Barriers	1–4	3.18	.50	−1.00	1.49	.81	1–4	3.11	.66	−1.11	.87	.85
Physical activity	33–9192	2216.74	1613.21	1.49	2.04	-	33–20424	3590.62*	2322.30	2.23	1.46	-

*Notes*: *M* = Mean; *S*D = Standard Deviation; S = Skewness; K = Kurtosis; IC = Internal Consistency coefficients; * statistically different at *p* < .05.

Several significant bivariate correlations emerged as theoretically expected (see Table 2): (a) PA was positively and significantly correlated with support provided by parents, best friend, and friends in both samples; (b) a similar trend was observed between perceived benefits and support provided by significant others; (c) perceived barriers displayed a negative or no significant association with PA and support provided by significant others; and (d) perceived benefits displayed a significant association with PA, with the correlation coefficient greater for boys compared to girls.

**Table 2.** Bivariate and Partial Correlations.

	Girls sample						Boys sample					
	1	2	3	4	5	6	1	2	3	4	5	6
1. Support best friend	1	.52**	.33**	.35**	−.23**	.22**	1	.56**	.33**	.31**	−.12*	.23**
2. Support friends	.53**	1	.27**	.33**	−.14*	.20**	.56**	1	.43**	.04	−.02	.22**
3. Support parents	.36**	.32**	1	.18**	.09	.10	.32**	.42**	1	.27**	.09	.17*
4. Benefits	.37**	.37**	.22**	1	−.13*	.23**	.30**	.37**	.32**	1	−.09	.29**
5. Barriers	−.25**	−.15*	.10	−.17*	1	.07	−.11*	−.05	.04	−.10	1	−0.5
6. Physical activity	.23**	.21**	.12*	.24**	.08	1	.26*	.25**	.18*	.30**	−.05	1

*Notes*: below diagonal line = bivariate correlations; above diagonal line = partial correlations controlling for age; *p < .05; **p < .01.

### Structural Equation Modelling Analyses

Before conducting structural equation modelling analysis, we performed a confirmatory factor analyses (CFA) of the proposed model to verify internal construct validity. We considered the traditional and incremental goodness-of-fit indexes a well as cutoffs as indications of validity reported in the method section. An a priori analysis revealed that missing values were less than .1% of the sample data for data that was completely missing at random levels in both male and female samples. Consequently, we relied on the full information maximum likelihood approach to handle missing data.

The CFA and structural equation models were performed using the maximum likelihood robust estimator against non-normal distribution data (as seen in Table 1). These models showed a good fit to the data in both boys and girls samples under analysis (see Table 3). Thus, we moved forward in analyzing the coefficients of the structural model. The standardized coefficients of the direct effects between the support provided by significant others and the perceived benefits, as well as, between the perceived benefits and PA were positive and significant in both models (see Table 4). In contrast, the standardized direct effects between the support provided by the best friend, friends, and parents and perceived PA barriers were not significant. Current results showed negative and significant associations between perceived barriers and PA. Explained PA variance was 24% and 33% for boys and girls, respectively.

**Table 3.** Goodness-of-Fit Indexes.

Models	χ^2^	df	CFI	TLI	SRMR	RMSEA	CI 90%
Girls sample							
Confirmatory model	662.27	363	.921	.916	.059	.067	.062–.074
Structural equation model	783.12	366	.915	.905	.077	.060	.052–.064
Boys sample							
Confirmatory model	581.91	363	.914	.910	.055	.062	.059–.066
Structural equation model	664.97	366	.909	.902	.070	.065	.061–.077

*Note*: SEM = Structural Equation Modelling; χ^2^ = Chi-square; df = degrees of freedom; CFI = Comparative Fit Index; TLI = Tucker Lewis Index; SRMR = Standardized Root Mean Square Residual; RMSEA = Root Mean Square Error of Approximation; CI90% = Confidence Interval at 90% for RMSEA.

**Table 4.** Structural Equation Model Analysis.

Regression paths	Direct		Regression paths	Indirect	
	β	CI 95%		β	CI 95%
Girls sample			Girls Sample		
Support friends → benefits	.30*	.11, .52	Mediator: Barriers		
Support friends → barriers	−.12	−.30, .11	Support friends → physical activity	.01	−.03, .11
Support friends → physical activity	.16*	.05, .35	Support best friend → physical activity	−.07	−.09, .23
Support best friend → benefits	.39*	.15, .57	Support parents → physical activity	−.08	−.10, .07
Support best friend → barriers	−.16	−.40, .05			
Support best friend → PA	.20*	.12, .53	Mediator: Benefits		
Support parents → benefits	.22*	.05, .40	Support friends → physical activity	.09*	.04, .17
Support parents → barriers	.11	−.15, .07	Support best friend → physical activity	.19*	.09, .51
Support parents → physical activity	.13*	.11, .30	Support parents → physical activity	.15*	.06, .29
Benefits → physical activity	.26*	.11, .43			
Barriers → physical activity	−.17*	−.31, −.14			
Boys sample			Boys sample		
Support friends → benefits	.29*	.09, .48	Mediator: Barriers		
Support friends → barriers	−.01	−.13, .13	Support friends → physical activity	.03	−.05, .011
Support friends → physical activity	.19*	.06, .43	Support best friend → physical activity	−.08	−.07, .24
Support best friend → benefits	.39*	.15, .57	Support parents → physical activity	−.11	−.15, .05
Support best friend → barriers	−.10	−.28, .13			
Support best friend → physical activity	.16*	.01, .28	Mediator: Benefits		
Support parents → benefits	.24*	.07, .38	Support friends → physical activity	.12*	.06, .19
Support parents → barriers	−.08	−.25, .11	Support best friend → physical activity	.17*	.07, .35
Support parents → physical activity	.09*	.09, .24	Support parents → physical activity	.10*	.02, .19
Benefits → physical activity	.23*	.06, .38			
Barriers → physical activity	−.09*	−.18, −.04			

*Notes*: β = standardized coefficients; CI95% = Confidence Interval at 95%; *p = level of significance at <.05.

### Mediation Analyses

Regarding the standardized indirect effects between support provided by significant others and PA levels, perceived benefits displayed a positive and significant indirect effect, but perceived barriers did not. To examine construct interactions on PA in more detail, mediation analyses were conducted considering only perceived benefits as a possible mediator in the relationship between support provided by significant others and PA. In Table 5, it is possible to observe the results from the mediation analyses of all support groups on PA for both girls and boys. Mediation appeared between support provided by the best friend and PA for girls (β = 0.14 [0.03−0.33]). Partial mediation was also observed between support provided by parents and PA for girls (β = 0.09 [0.04−0.37]). Total indirect effects were significant in all tested models, showing a partial mediation effect.

**Table 5.** Mediation Analysis.

Direct regression paths	β	CI 95%	Indirect regression paths	β	CI 95%	Total indirect paths	β	CI 95%
Girls sample								
Independent variable: Support friends								
Support friends → physical activity	.18	.02, .22	Support friends → benefits	.51	.34, .68	Support friends → benefits → physical activity	.13	.02, .29
			Benefits → physical activity	.25	.07, .43			
Independent variable: Support best friend								
Support best friends → physical activity	.11	.02, .15	Support best friend → benefits	.64	.45, .83	Support best friend → benefits → physical activity	.14	.03, .33
			Benefits → physical activity	.22	.06, .39			
Independent variable: Support parents								
Support parents → physical activity	.08	.01, .20	Support parents → benefits	.38	.18, .57	Support parents → benefits → physical activity	.07	.01, .18
			Benefits → physical activity	.18	.05, .33			
Boys sample								
Independent variable: Support friends								
Support friends → physical activity	.24	.14, .29	Support friends → benefits	.50	.34, .67	Support friends → benefits → physical activity	.11	.05, 40
			Benefits → physical activity	.22	.15, .60			
Independent variable: Support best friend								
Support best friend → physical activity	.31	.03, .60	Support best friend → benefits	.48	.28, .68	Support best friend → benefits → physical activity	.10	.05, 40
			Benefits → physical activity	.20	.17, .59			
Support parents → physical activity	.08	.05, .34	Support parents → benefits	.48	.29, .67	Support parents → benefits → physical activity	.09	.04, 37
			Benefits → physical activity	.19	.13, .55			

*Notes*: β = standardized coefficients; CI95% = Confidence Interval at 95%.

## Discussion

In this study we aimed to investigate whether the relationship between social support provided to adolescents by significant others and the PA levels of adolescents was mediated by the adolescents’ perceived barriers and benefits of PA. Examining the factors that influence adolescents’ PA is of paramount importance as these individuals tend to decrease their physically active behaviors as they transition to adulthood. To the best of our knowledge, this was the first study of the association between social support provided by three significant groups and PA among adolescents, considering the mediation role of perceived PA barriers and benefits during this key developmental period. Our results displayed significant associations between support provided by significant others and PA in both boys and girls, with predictor variables showing 24% and 33% of explained PA variance for these subgroups, respectively.

### Associations Between Perceived Support, Benefits, and Barriers of PA

For girls, support for PA provided by the best friends, displayed a more significant association with perceived benefits of PA, compared to support provided by friends and parents. For boys, the most significant association was between support provided by friends and perceived benefits of PA. Results tend to support results from previous studies examining the role of the best friend in PA promotion (Lopes et al., 2013, 2015; King et al., 2008; Monteiro et al., 2021). For example, among adolescents who participate in sports, friends were a strong motive for their PA involvement (Coleman et al., 2008). Differences between girls and boys regarding the most significant support could be related to the relationships built during adolescence. Girls have seemed to be more prone to be physically active if there is a significant friendship that influenced them to engage in PA (Lopes et al., 2015). Among boys, there is some information about the particular influence of the best friend as a function of modeling PA, by co-participation and encouragement, to show the benefits of regular PA (Monteiro et al., 2021). Results from this study suggest that role modelling, support, encouragement and praise to explain the benefits of regular PA acted as positive influences in promoting PA. Previous research within the ecological model also explained the importance of significant others throughout the lifespan, as friends and specifically best friends are highly valued influnces during adolescence (Stokols, 1992). The associations between support provided by significant others and perceived barriers were, in general, non-significant or negatively correlated.

### Perceived Barriers and Benefits of PA and Levels of PA

In general, we found that perceived PA benefits were positively associated with PA levels in both groups. These results align with previous studies (Bélanger et al., 2011; Martins et al., 2015; Roth et al., 2019) suggesting that adolescent boys and girls who perceive benefits of PA, such as physical appearance, social interactions, positive experiences, and enjoyment, recognize its importance and show higher levels of PA. In contrast, we found that the relationships between perceived barriers of PA and PA levels tended to be negative, supporting existing literature showing that non-enjoyment of PA, boredom, lack of confidence in the ability to be physically active, and insufficient time to be active related to reduced PA engagement (Bassett-Gunter et al., 2017; Bélanger et al., 2011; Berntsson & Ringsberg, 2014; Hsu et al., 2011). Perceived barriers among youth such as time constraints, weak motivation levels, perceived incompetence, excessive costs, and limited access or lack of places to practice led to greater perceptions of these barriers and lower amounts of PA.

### Mediating Role of Perceived Benefits of PA

One of the novelties of this study was our measurement and analysis of the mediation role of perceived benefits and barriers in the relationship between support provided by three groups of significant others and adolescents’ levels of PA. We did not find perceived barriers to mediate this relationship, as indirect paths were non-significant (*p* < .05). However, perceived benefits displayed an indirect mediating effect. In sum, perceived PA benefits partially mediated the relationship between perceived support provided by significant others and PA engagement. For girls, while total indirect coefficients (Table 5) were not significant, the support provided by the best friend was the most significant association. For boys, the support provided by friends was the most significant contributor in the mediation analysis. These results show how central the best friend is for adolescent girls in promoting PA. As shown by Jago et al. (2011), adolescent girls who frequently engage in PA with their best friend obtain higher PA levels, as they perceive benefits from doing it together, with each friend motivateing the other to continue long-term PA. Boys who take part in PA with their friends at home or in a sports club show higher amounts of PA. Nonetheless, support provided by parents also displayed a positive and significant indirect association with PA. Hence, both parents and peers have a meaningful role in shaping PA among adolescents (Smith, 2019). Therefore as stated by Horn (2019), several social factors likely contribute to PA levels during adolescence. Jago et al. (2011) did not find differences between boys and girls in social support from best friends, although their sample was comprised of younger adolescents, aged 10–11-years old. On the other hand, Stearns et al. (2019) found that girls’ best friends exhibited more similar levels of overall PA than their non-friends and that, for boys, only reciprocated friends had similar levels of PA, compared to unreciprocated friendships. These associations need to be explored in the future in terms of intensity, frequency, and type (i.e., physical education, leisure-time physical activity).

Our results reinforced the idea that support provided by significant others with which adolescents have daily contact help determine the interaction between perceived PA benefits and PA engagement. Our results provided new insight by assessing the role of support of more than just one group of significant others and better understanding their correlations with PA (e.g., Howie et al., 2018; Hsu et al., 2011; Martins et al., 2015) and perceived PA benefits (e.g., Roth et al., 2019). Having adolescents model and spend time being active together are important ways to continually capitalize on the influence of friendship between best friends (Jago et al., 2011). In addition, as stated by De La Haye et al. (2011), resemblances in amount of PA may not be a permanent characteristic of friendship, as it is not clear whether these differences or similarities are due to friendship selection or friendship influence. Our results align with previous findings (Lopes et al., 2013, 2015) suggesting that, regardless of sex, having a best friend who also engages in PA positively influences increased PA.

A curious aspect of our findings was that the amount of total variance of PA engagement that was explained by the models in adolescent boys and girls was 24% and 43%, respectively. Examining the paths of each model in each sub-sample (see Table 4 for more details), adolescent girls’ perceived support and benefits impacted their PA more than these factors impacted boys’ PA. This finding was partly supported by previous studies (Monteiro et al., 2021; Stearns et al., 2019) and it highlights, particularly, the importance of the best friend for promoting PA among adolescent girls. Understanding these influences is particularly important for girls, because they have been consistently found to be less active than boys (Laird et al., 2016). Among various types of social support, our results support the notion that, for boys, parents and friends may have a greater role in enhancing PA. Again, delineating these subtle correlates and possible determinants of PA in adolescent boys and girls is critically important to developing future interventions for promoting healthy PA behaviors.

### Strengths, Limitations, and Directions for Future Research

A major strength of this study was the assessment of support provided to adolescents by three different groups of significant others. However, our participants were recruited from a single country, possibly limiting our ability to extrapolate findings to other cultures and contexts. Additionally, we used self-report measures and metabolic equivalents to measure PA and these may not be directly comparable to findings in previous research. Future investigators should include a larger and more diverse participant sample and should use objective measures (e.g., accelerometer), especially regarding PA.

## Conclusion

Our findings in this study of adolescents provide evidence of the criterion-related validity and reliability of social support for promoting PA, and we detailed the sources of social support from significant others that may be most important. Our study was the first to identify the differential influence of support provided by the best friend, friends, and parents for boys and girls, while considering their roles as mediators of the interactions between perceived barriers and benefits of PA. Future prospective research with longitudinal designs should be conducted to (a) evaluate the predictive role of support provided by significant others and (b) demonstrate the efficacy of varied means of enhancing PA in youth.
